# Patient Sociodemographics and Comorbidities and Birth Hospital Characteristics Associated With Postpartum Emergency Department Care

**DOI:** 10.1001/jamanetworkopen.2023.3927

**Published:** 2023-03-21

**Authors:** Haley Zarrin, Carmen Vargas-Torres, Teresa Janevic, Toni Stern, Michelle P. Lin

**Affiliations:** 1 Department of Emergency Medicine, Icahn School of Medicine at Mount Sinai, New York, New York; 2 Department of Population Health Science and Policy, Icahn School of Medicine at Mount Sinai, New York, New York; 3 Department of Obstetrics, Gynecology, and Reproductive Science, Icahn School of Medicine at Mount Sinai, New York, New York; 4 Department of Emergency Medicine, Stanford University, Palo Alto, California

## Abstract

**Importance:**

Postpartum emergency department (ED) visits may indicate poor access to care and risk for maternal morbidity.

**Objectives:**

To identify patient and hospital characteristics associated with postpartum ED visit rates.

**Design, Setting, and Participants:**

This retrospective cohort study used data from the 2014 to 2016 New York State Inpatient Database and State Emergency Department Database. All obstetric discharges from acute care hospitals in New York State from January 1, 2014, through November 15, 2016, were included. Obstetric discharges in the inpatient database were linked to subsequent ED visits by the same patient in the ED database. Data were analyzed from February 2020 to August 2022.

**Exposures:**

Patient characteristics assessed included age, race, insurance, home zip code income quartile, Charlson Comorbidity Index score, and obstetric risk factors. Hospital characteristics assessed included safety net status, teaching status, and status as a hospital disproportionally serving racial and ethnic minority populations.

**Main Outcomes and Measures:**

The primary outcome was any ED visit within 42 days of obstetric discharge. Multilevel logistic regression with 2-level nested mixed effects was used to account for patient and hospital characteristics and hospital-level clustering.

**Results:**

Of 608 559 obstetric discharges, 35 299 (5.8%) were associated with an ED visit within 42 days. The median (IQR) birth hospital postpartum ED visit rate was 6.3% (4.6%-8.7%). The mean (SD) age was 28.4 (9.1) years, 53 006 (8.7%) were Asian patients, 90 675 (14.9%) were Black patients, 101 812 (16.7%) were Hispanic patients, and 275 860 (45.3%) were White patients; 292 991 (48%) were insured by Medicaid, and 290 526 (47.7%) had private insurance. Asian patients had the lowest postpartum ED visit rates (2118 ED visits after 53 006 births by Asian patients [3.99%]), and Black patients had the highest postpartum ED visit rates (8306 ED visits after 90 675 births by Black patients [9.15%]). Odds of postpartum ED visits were greater for Black patients (odds ratio [OR], 1.31; 95% CI, 1.26-1.35; *P* < .001) and Hispanic patients (OR, 1.19; 95% CI, 1.15-1.24; *P* < .001) relative to White patients; those with Medicare (OR, 1.55; 95% CI, 1.39-1.72; *P* < .001), Medicaid (OR, 1.37; 95% CI, 1.34-1.41; *P* < .001), or self-pay insurance (OR, 1.50; 95% CI, 1.41-1.59; *P* < .001) relative to commercial insurance; births that occurred at safety net hospitals (OR, 1.43; 95% CI, 1.37-1.51; *P* < .001) and hospitals disproportionately serving racial and ethnic minority populations (OR, 1.14; 95% CI, 1.08-1.20; *P* < .001); and births that occurred at hospitals with fewer than 500 births per year (OR, 1.25; 95% CI, 1.14-1.39; *P* < .001) relative to those with more than 2000 annual births. Adjusted odds of postpartum ED visits were lower after birth at teaching hospitals (OR,  0.82; 95% CI, 0.74-0.91; *P* < .001) and metropolitan hospitals (OR, 0.74; 95% CI, 0.65-0.85; *P* < .001).

**Conclusions and Relevance:**

This cohort study found that Black and Hispanic patients experienced higher adjusted odds of postpartum ED visits across all hospital types, particularly at safety net hospitals and those disproportionately serving racial and ethnic minority populations . These findings support the urgent need to mitigate structural racism underlying maternal health disparities.

## Key Points

**Question:**

Which patient and hospital characteristics are associated with postpartum ED visits?

**Findings:**

This retrospective cohort study including 608 559 obstetric discharges found higher odds of postpartum ED visits among patients who were younger, from racial and ethnic minority groups, publicly insured, and had more comorbid medical conditions. Postpartum ED visits were more likely after giving birth at hospitals serving racial and ethnic minority groups, safety net hospitals, and hospitals with fewer obstetric discharges.

**Meaning:**

These findings suggest that Black and Hispanic patients experienced higher rates of postpartum ED visit rates across all hospital types, particularly at safety net hospitals and those serving racial and ethnic minority groups, suggesting structural changes are needed to reduce maternal health disparities.

## Teaser

This cohort study examines patient and hospital characteristics associated with postpartum emergency department visits in New York State from January 1, 2014, to November 15, 2016.

## Introduction

US maternal mortality rates doubled between 1991 and 2014, and most maternal deaths occurred postpartum.^1^ Ongoing attention to racial disparities in health has spurred research about the differences in maternal morbidity and mortality across various populations in the US. Although risk factors for severe maternal morbidity have been identified,^2^ people who give birth continue to experience increasingly high rates of poor postpartum outcomes in the US.^1^ Emergency department visits during the postpartum period are potentially preventable and can be a sentinel event for patients at increased risk of morbidity. Prior research has suggested that patients from racial and ethnic minority groups identify access to care and communication with their medical team as key factors in reducing the likelihood of postpartum ED visits.^3^ Interventions to improve communication and care coordination are limited by the lack of knowledge regarding which hospital settings should be targeted for these interventions. Single-institution studies have examined the incidence and reasons for emergency department visits and ICU admissions among pregnant and postpartum patients.^4,5,6,7,8,9,10,11^ However, it is unknown whether these findings are generalizable across institutions and populations and to what extent hospital factors contribute to potentially avoidable postpartum ED visits. Therefore, we use a statewide data set to describe the patient and birth hospital characteristics associated with postpartum ED visits within 42 days of giving birth, including racial distribution by hospital type and most common diagnoses associated with postpartum ED visits.

## Methods

This cross-sectional study used the Strengthening the Reporting of Observational Studies in Epidemiology (STROBE) reporting guideline for reporting observational studies. Informed consent was waived because this study used publicly available, deidentified data, and the institutional review board at Icahn School of Medicine at Mount Sinai deemed the study exempt from review.

We conducted a cross-sectional study of all obstetric discharges from January 1, 2014, to November 15, 2016, and subsequent ED visits (through December 30, 2016) by the same patient in New York State by linking the State Inpatient Database to the State Emergency Department Database. Obstetric discharges were identified using the *International Classification of Diseases, Ninth Revision *(*ICD-9*) (January 1, 2014, through September 30, 2015) and *ICD-10* (October 1, 2015, through December 31, 2016) diagnosis and procedure codes that have previously been validated for use in administrative data sets.^12,13^

We focused on ED visits within 42 days of obstetric discharge because guidelines in effect during the study period recommended an initial postpartum visit at 42 days.^14^ We calculated the postpartum ED visit rate as the proportion of patients with at least 1 ED visit to any hospital who had an obstetric discharge within the preceding 42 days. We also calculated the hospital-level postpartum ED visit rate, defined as the proportion of patients with an ED visit to any hospital who had an obstetric discharge from a given index hospital within the preceding 42 days. We report the primary or first-listed *ICD-9* and *ICD-10* codes associated with the first ED visit occurring within the study period.

Maternal characteristics included age, race, insurance, and home zip code income quartile. Because prior research and National Institutes of Health have identified structural racism as a key factor in maternal morbidity,^15^ we measured postpartum ED visit rates stratified by race and hospital type. Race and ethnicity data were obtained from existing administrative data, which was self-reported when available, and has previously been validated to have high concordance with self-reported vital statistics data.^16^ We define race as a social construct, and we use it as a stratification variable as a proxy for structural racism or the totality of exposures due to racism and oppression.^17^ We ascertained race and ethnicity using the single combined variables as reported by the Health Care Utilization Project databases, which is collected from hospital discharge records.^18^ We accounted for nonobstetric risk by adjusting for comorbid medical conditions using the previously validated Charlson Comorbidity Index while also including dummy variables for pre-existing diabetes, hypertension, asthma, and obesity, which are not captured in the Charlson score. Additional variables included obstetric risk factors, including preeclampsia, eclampsia, gestational diabetes, diabetes, gestational hypertension, hypertension, asthma, obesity, multiple gestations, substance use disorder, mental health disorder, and prior cesarean birth. Encounters with missing age, race or ethnicity, and/or income were dropped (8515 encounters or 1.38% of sample).

Variables associated with severe maternal morbidity, as per the Centers for Disease Control and Prevention (CDC)^2^ were defined as perinatal complications for all birth types, including vaginal, cesarean, and operative vaginal. We tested model versions including individual dummy variables for each condition from the CDC list, as well as a combined variable (severe maternal morbidity) for the presence of any condition appearing on the CDC list, and elected to use the latter within the final model for parsimony. Blood transfusion was retained as a separate indicator variable and not combined within the severe maternal morbidity indicator variable as per prior studies.^19^

We defined hospital characteristics associated with the index birth, including hospitals disproportionately serving racial and ethnic minority populations (defined as the top quartile of hospitals with respect to the overall proportion of all-cause discharges of Black and Hispanic patients, hereafter referred to as minority-serving); safety net status (defined as the top quartile of hospitals with respect to the proportion of Medicaid and uninsured discharges); teaching status as defined by the American Hospital Association; metropolitan vs nonmetropolitan location as defined by Core-based Statistical Areas; and obstetric volume (measured as total annual obstetric discharges). Additionally, we calculated the ratio of obstetric discharges to all hospital discharges to quantify the extent to which obstetric services account for each hospital’s inpatient care. We excluded births at hospitals with fewer than 200 obstetric discharges per year (5121 [0.84%]) due to small cell sizes resulting in significant variance and outliers in hospital-level ED revisit rates. For example, 38 hospitals had postpartum ED visit rates of more than 90%, and 15 had no postpartum ED visits.

### Statistical Analysis

Descriptive statistics were performed using parametric (*t* tests) and nonparametric (χ^2^) tests as appropriate for all patient and hospital factors. We performed multilevel logistic regression with a 2-level nested mixed effects to account for clustering at the hospital level to determine predictors of ED visits within 42 days of obstetric discharge after adjusting for patient and index hospital characteristics. We first fit the model using patient characteristics and then sequentially added each hospital-level variable, using likelihood ratio tests to compare model fit.

Index births resulting in death or transfer were excluded. All analyses were conducted using Stata version SE16.0 (StataCorp) from January 2020 through July 2022. All hypothesis tests were 2-sided, and statistical significance was set at *P* < .05. Data were analyzed from February 2020 to August 2022.

## Results

There were 608 559 eligible births identified with a mean (SD) age 28.4 (9.1) years, of which 53 006 (8.7%) were Asian individuals, 90 675 (14.9%) were Black individuals, 101 812 (16.7%) were Hispanic individuals, 275 860 (45.3%) were White individuals, 292 991 (48.1%) were insured by Medicaid, and 290 526 (47.7%) had private insurance (Table 1). We identified 35 299 (5.8%) births associated with at least 1 ED visit within 42 days; 5218 (0.9%) were associated with 2 or more ED visits within 42 days; and 5295 (0.9%) were associated with readmission from the ED. The median (IQR) hospital-level postpartum ED visit rate was 6.3% (4.6%-8.7%).

**Table 1.** Patient and Hospital Characteristics of Obstetric Discharges in New York State, 2014-2016

Characteristic	No. (%)		
	ED visit within 42 d	No ED visit within 42 d	Total sample
No. (%)	35 299 (5.8)	573 260 (94.2)	608 559 (100)
Age, y^a^			
<20	3297 (9.3)	49 404 (8.6)	52 701 (8.7)
20-24	6520 (18.5)	83 669 (14.6)	90 189 (14.8)
25-29	9015 (25.5)	142 272 (24.8)	151 287 (24.9)
30-34	8832 (25)	169 050 (29.5)	177 882 (29.2)
35-39	5475 (15.5)	100 110 (17.5)	105 585 (17.4)
≥40	2164 (6.1)	28 751 (5.0)	30 914 (5.1)
Race and ethnicity^a^			
Asian	2118 (6.0)	50 888 (8.9)	53 006 (8.7)
Black	8306 (23.5)	82 369 (14.4)	90 675 (14.9)
Hispanic	7353 (20.8)	94 459 (16.5)	101 812 (16.7)
White	12 514 (35.5)	263 346 (45.9)	275 860 (45.3)
Native American and other^b^	5009 (14.2)	82 198 (14.3)	87 207 (14.3)
Insurance^a^			
Medicare	508 (1.4)	2778 (0.47)	3286 (0.5)
Medicaid	22 041 (62.4)	270 950 (47.3)	292 991 (48.1)
Private insurance	11 169 (31.6)	279 357 (48.7)	290 526 (47.7)
Self pay	932 (2.6)	10 874 (1.9)	11 806 (1.9)
No charge	14 (0.04)	193 (0.03)	207 (0.03)
Other	654 (1.9)	9934 (1.7)	10 489 (1.7)
Income quartile^a^			
1 (lowest)	11 331 (32.1)	150 669 (26.3)	162 000 (26.6)
2	9481 (26.9)	147 466 (25.7)	156 947 (25.8)
3	8159 (23.2)	138 625 (24.2)	146 784 (24.1)
4 (highest)	6184 (17.5)	128 186 (22.4)	134 370 (22.1)
Charlson comorbidity score^a^			
0	8691 (24.6)	289 503 (50.5)	298 194 (49.0)
1 or 2	11.995 (34)	184 448 (32.2)	196 443 (32.3)
≥3	14 614 (41.4)	99 308 (17.3)	113 922 (18.7)
Medical history^a^			
Diabetes, nongestational	1045 (3.0)	5467 (1.0)	6512 (1.1)
Hypertension	3219 (8.8)	9134 (2.7)	12 354 (3.1)
Asthma	5090 (14.4)	38 544 (6.7)	43 634 (7.2)
Obesity	6078 (17.2)	49 544 (8.6)	55 622 (9.1)
Birth hospital characteristics			
Minority serving hospital^a^	13 498 (38.2)	152 030 (26.5)	165 529 (27.2)
Safety net hospital^a^	14 716 (41.7)	163 348 (28.5)	178 064 (29.3)
Teaching hospital^a^	12 672 (35.9)	250 773 (43.9)	263 445 (43.3)
Metropolitan hospital	33 252 (94.2)	548 652 (95.7)	581 904 (95.6)
Proportion of obstetric discharges to all birth hospital discharges, mean (SD) %	3.2 (1.9)	4.3(5.3)	4.2 (5.1)
Annual obstetric discharges^a^			
≤500	2210 (6.3)	21 862 (3.8)	24 071 (4.0)
501-2000	14 095 (39.9)	187 695 (32.7)	201 790 (33.2)
≥2001	18 994 (53.8)	363 703 (63.5)	382 697 (62.9)

Abbreviation: ED, emergency department.

^a^ All values were significantly different (*P* < .01) between groups with and without postpartum ED visit within 42 days. There were 120, 117, and 123 hospitals included in the sample in 2014, 2015, and 2016, respectively, with 123 hospitals appearing in the data set in any year.

^b^ Includes Native American individuals and patients who self-designated their race as other.

Postpartum ED visits were more frequent among births by patients who were younger than 30 years at delivery, identified as Black (8306 [23.5%] with an ED visit vs 82 369 [14.4%] without) or Hispanic (7353 [20.8%] with an ED visit vs 94 459 [16.5%] without), covered by Medicaid insurance (22 041 [62.4%] with an ED visit vs 270 950 [47.3%]), and from the lowest and second lowest income quartiles (11 331 [32.1%] and 9481 [26.9%] with an ED visit vs 150 669 [26.3%] and 147 466 [25.7%] without, respectively). Postpartum ED visits were less frequent among patients who identified as White individuals (12 514 [35.5%] with an ED visit vs 263 346 [45.9%] without), were commercially insured (11 169 [31.6%] with an ED visit vs 279 357 [48.7%] without), and who lived in the top quartile of income zip codes (6184 [17.5%] with an ED visit vs 128 186 [22.4%] without).

All births with the obstetric characteristics and medical history described in Table 2 were significantly more likely to be associated with a postpartum ED visit, and patients with a higher Charlson comorbidity score. In addition, patients with the condition that qualified as severe maternal morbidity, and those who received transfused blood products were significantly more likely to have a postpartum ED visit.

**Table 2.** Clinical Factors Associated With Index Obstetric Discharge

Clinical factors	No. (%)		
	ED visit within 42 d	No ED visit	Totals
No.	35 299 (5.8)	573 260 (94.2)	608 559
Obstetric characteristics			
Preeclampsia	2280 (6.5)	18 350 (3.2)	20 630 (3.4)
Gestational diabetes	3512 (10.0)	43 955 (7.7)	47 468 (7.8)
Gestational hypertension	1355 (3.8)	14 832 (2.6)	16 188 (2.7)
Multiple gestation	964 (2.7)	11 086 (1.9)	12 049 (2.0)
Maternal substance use	727 (2.1)	4993 (0.9)	5720 (0.9)
Maternal mental health	3865 (11.0)	30 153 (5.3)	34 018 (5.6)
Prior cesarean birth	7780 (22.0)	97 014 (16.9)	104 794 (17.2)
Birth characteristics^a^			
Operative vaginal	3897 (11.0)	20 324 (3.5)	24 221 (4.0)
Cesarean	15 599 (44.2)	180 966 (31.6)	196 595 (32.3)
Premature	2714 (7.7)	30 391 (5.3)	33 106 (5.4)
Severe maternal morbidity^a^^,^^b^	3992 (7.4)	19 098 (1.5)	22 091 (1.8)
Acute myocardial infarction	16 (0)	160 (0)	176 (0)
Aneurysm	7 (0)	54 (0)	61 (0)
Acute renal failure	441 (1.3)	1141 (0.2)	1582 (0.3)
Adult respiratory distress syndrome	445 (1.3)	1016 (0.2)	1461 (0.2)
Amniotic fluid embolism	4 (0)	0	4 (0)
Cardiac arrest/ventricular fibrillation	71 (0.2)	112 (0)	183 (0)
Conversion of cardiac rhythm	35 (0.1)	147 (0)	183 (0)
Disseminated intravascular coagulation	219 (0.6)	1181 (0.2)	1400 (0.2)
Eclampsia	445 (1.3)	468 (0.1)	913 (0.2)
Heart failure/arrest during surgery or procedure	14 (0)	108 (0)	609 (0)
Puerperal cerebrovascular disorders	180 (0.5)	429 (0.1)	609 (0.1)
Pulmonary edema or acute heart failure	406 (1.2)	507 (0.1)	913 (0.2)
Severe anesthesia complications	21 (0.1)	101 (0)	122 (0)
Sepsis	1031 (2.9)	2377 (0.4)	3408 (0.6)
Shock	244 (0.7)	730 (0.1)	974 (0.1)
Sickle cell disease with crisis	67 (0.2)	176 (0)	243 (0)
Air or thrombotic embolism	258 (0.7)	533 (0.1)	791 (0.1)
Hysterectomy	215 (0.6)	880 (0.2)	1095 (0.2)
Temporary tracheostomy	21 (0.1)	40 (0)	61 (0)
Ventilation	318 (0.9)	1569 (0.3)	1887 (0.3)
Blood products transfusion	1079 (5.9)	11 735 (2.0)	13 814 (2.3)

Abbreviation: ED, emergency department.

^a^ All values were significantly different (*P* < .01) between groups with and without postpartum ED visit within 42 days with the exception of amniotic fluid embolism (*P* = .08).

^b^ Severe maternal morbidity was identified using the US Centers for Disease Control and Prevention definition.^2^

Postpartum ED visits were more likely to occur after births at minority-serving hospitals (13 498 [38.2%] with an ED visit vs 152 030 [26.5%] without), safety net hospitals (14 716 [41.7%] with an ED visit vs 163 348 [28.5%] without), and small- to medium-sized hospitals (2210 [6.3%] and 14 095 [39.9%] with an ED visit vs 21 862 [3.8%] and 187 695 [32.7%] without, respectively). Postpartum ED visits were less likely to occur after births at large hospitals (18 994 [53.8%] with an ED visit vs 363 703 [63.5%] without), teaching hospitals (12 672 [35.9%] with an ED visit vs 250 773 [43.9%] without), and hospitals in metropolitan areas (33 252 [94.2%] with an ED visit vs 548 652 [95.7%] without).

**Figure 1.**
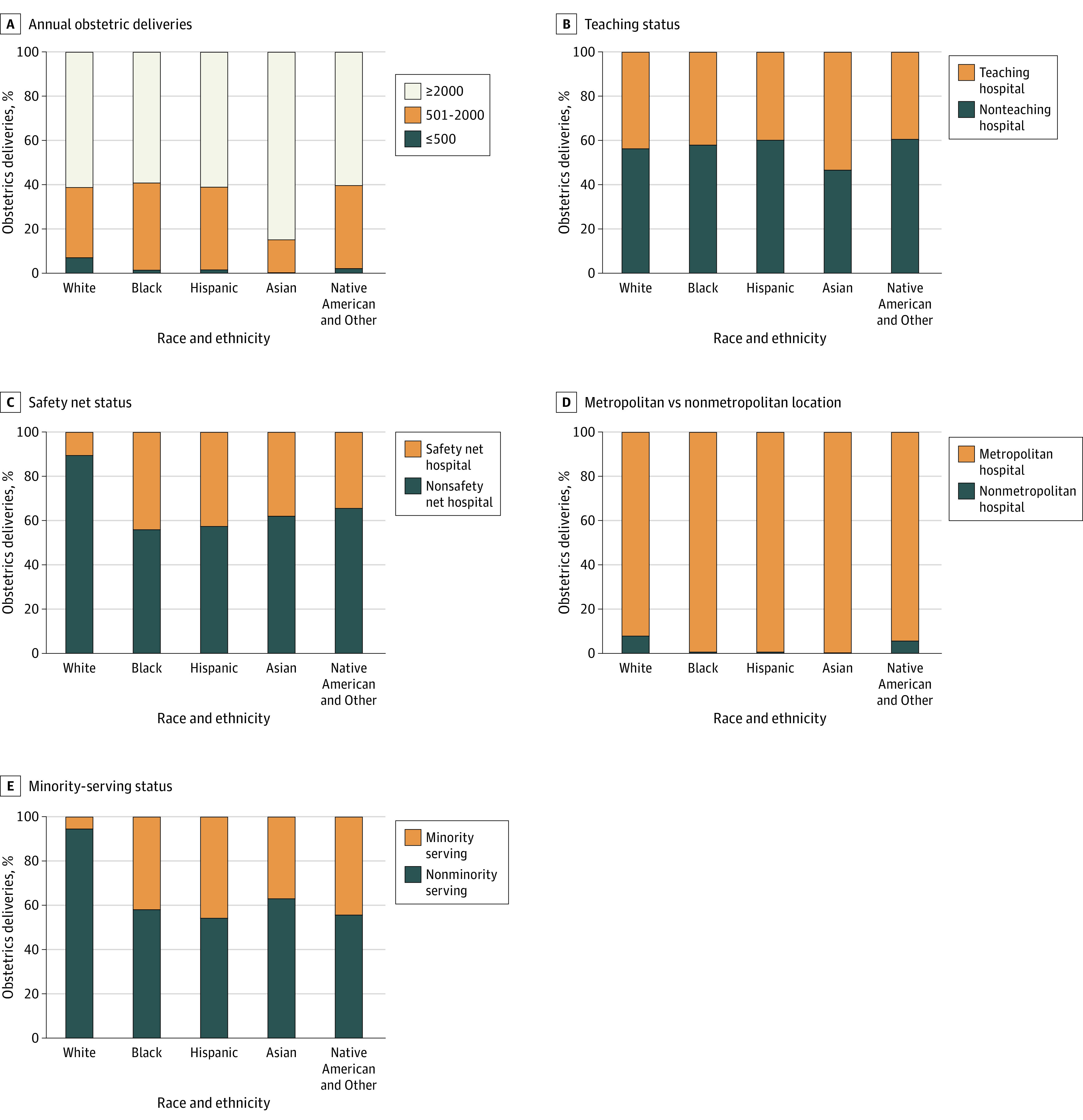
Distribution of Birth Hospital Types by Race and Ethnicity

**Figure 2.**
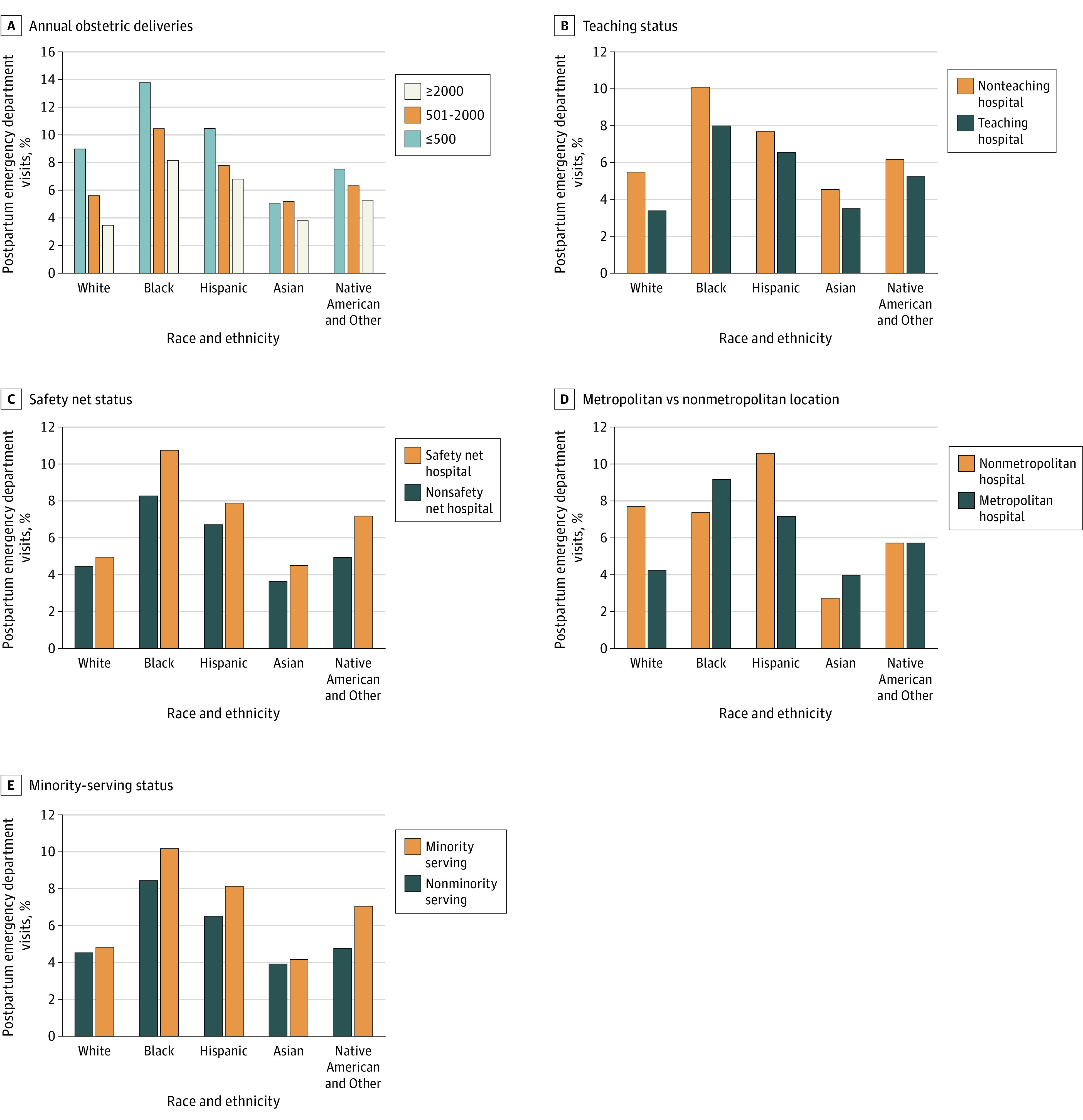
Birth Hospital Postpartum Emergency Department Visit Rates by Race and Hospital Type

With respect to the racial distribution of patients across hospital types, the proportion of White patients with index obstetric discharges at safety net (20 414 [10.3%]) and nonminority-serving (14 157 [5.5%]) hospitals were the lowest of any racial group. Other racial distributions across hospital types are shown in Figure 1. Across all hospital types, Asian patients had the lowest postpartum ED visit rates, and Black patients had the highest postpartum ED visit rates (with the exception of nonmetropolitan hospitals, at which Hispanic patients had the highest postpartum ED visit rate) as per Figure 2.

After adjusting for patient and hospital characteristics (Table 3), patients had higher adjusted odds of postpartum ED visits if they were younger than 20 years (odds ratio [OR], 1.59; 95% CI, 1.51-1.66; *P* < .001), 20 to 24 years (OR, 1.18; 95% CI, 1.14-1.22; *P* < .001), or 25 to 29 years (OR, 1.05, 95% CI, 1.02-1.09; *P* < .001) compared with those age 30 to 34 years, Black patients (OR, 1.31; 95% CI, 1.26-1.35; *P* < .001), Hispanic patients (OR, 1.19; 95% CI, 1.15-1.24; *P* < .001), or Native American/other race patients (OR, 1.12; 95% CI, 1.08-1.17; *P* < .001) relative to White patients, being insured by Medicare (OR, 1.55; 95% CI, 1.39-1.72; *P* < .001), Medicaid (OR, 1.37; 95% CI, 1.34-1.41; *P* < .001), or self-pay (OR, 1.50; 95% CI, 1.41-1.59; *P* < .001) relative to commercial insurance, and if they had more preexisting comorbid chronic conditions (Charlson comorbidity score of 1 to 2 [OR, 1.76; 95% CI, 1.71-1.82; *P* < .001] or more than 3 [OR, 3.03; 95% CI, 2.93-3.13; *P* < .001) relative to those without comorbidities). Those with incomes in the lowest income quartile (OR, 0.9; 95% CI, 0.89-0.97, *P* < .001) and second lowest quartile (OR, 0.94; 95% CI, 0.91-0.98; *P* = .001) had lower adjusted odds of postpartum ED visits relative to those in the top quartile.

**Table 3.** Adjusted Odds of Patient and Hospital Characteristics Associated With ED Visits Within 42 Days of Obstetric Discharge

Characteristics	Unadjusted postpartum ED visit rate	Adjusted OR (95% CI)	*P* value
Age, y			
<20	6.25	1.59 (1.51-1.66)	<.001
20-24	7.22	1.18 (1.14-1.22)	<.001
25-29	5.96	1.05 (1.02-1.09)	<.001
30-34	4.96	1 [Reference]	NA
35-39	5.19	0.99 (0.96-1.03)	.86
≥40	7.00	1.05 (1.00-1.11)	.05
Race			
Asian	3.99	1.03 (0.98-1.09)	.31
Black	9.15	1.31 (1.26-1.35)	<.001
Hispanic	7.23	1.19 (1.15-1.24)	<.001
White	4.53	1 [Reference]	NA
Native American and other^a^	5.75	1.12 (1.08-1.17)	<.001
Insurance			
Private	2.84	1 [Reference]	NA
Medicare	15.98	1.55 (1.39-1.72)	<.001
Medicaid	7.52	1.37 (1.34-1.41)	<.001
Self pay	7.25	1.50 (1.41-1.59)	<.001
Income quartile			
1 (lowest)	6.99	0.93 (0.89-0.97)	<.001
2	6.04	0.94 (0.91-0.98)	.001
3	5.58	0.97 (0.93-1.00)	.07
4 (highest)	4.61	1 [Reference]	NA
Charlson comorbidity score			
0	2.91	1 [Reference]	NA
1-2	6.11	1.76 (1.71-1.82)	<.001
≥3	12.82	3.03 (2.93-3.13)	<.001
Medical history			
Diabetes	16.1	1.02 (0.94-1.10)	.82
Hypertension	16.4	1.67 (1.59-1.75)	<.001
Asthma	11.7	1.10 (1.07-1.14)	<.001
Obesity	10.9	1.16 (1.12-1.20)	<.001
Obstetric risk factors			
Preeclampsia	11.1	1.21 (1.15-1.27)	<.001
Gestational diabetes	7.4	0.96 (0.92-1.00)	.05
Gestational hypertension	8.4	1.16 (1.09-1.23)	<.001
Multiple gestation	8	0.93 (0.87-1.00)	.06
Maternal substance use	12.7	0.98 (0.90-1.06)	.64
Maternal mental health	11.4	1.32 (1.27-1.37)	<.001
Prior cesarean birth	7.4	0.86 (0.83-0.88)	<.001
Birth characteristics			
Operative vaginal	16.1	1.83 (1.76-1.91)	<.001
Cesarean	7.93	1.47 (1.43-1.52)	<.001
Premature	8.2	1.05 (1.00-1.10)	.04
Severe maternal morbidity			
Any SMM except transfusion	23.8	2.57 (2.44-2.70)	<.001
Blood products transfusion	15.1	1.43 (1.35-1.51)	<.001
Hospital characteristics			
Minority serving hospital	7.72	1.14 (1.08-1.20)	<.001
Safety net hospital	7.42	1.43 (1.37-1.51)	<.001
Teaching hospital	4.81	0.82 (0.74-0.91)	<.001
Metropolitan hospital	5.71	0.74 (0.65-0.85)	<.001
Proportion of obstetric discharges to all hospital discharges	n/a	0.92 (0.91-0.94)	<.001
Annual obstetric discharges			
≤500	9.20	1.25 (1.14-1.39)	<.001
501-2000	6.98	1.04 (1.00-1.10)	.08
≥2001	4.96	1 [Reference]	NA
Year			
2014	6.06	1 [Reference]	NA
2015	6.05	0.94 (0.92-0.97)	<.001
2016	5.87	1.07 (1.04-1.10)	<.001

Abbreviations: ED, emergency department; OR, odd ratio; SMM, severe maternal morbidity.

^a^ Includes Native American and patients who self-designated their race as other.

Obstetric characteristics associated with higher adjusted odds of postpartum ED visits were cesarean birth (OR, 1.47; 95% CI, 1.43-1.52; *P* < .001), maternal mental health diagnosis (OR, 1.32; 95% CI, 1.27-1.37, *P* < .001), operative vaginal birth (OR, 1.83; 95% CI, 1.76-1.91; *P* < .001), preeclampsia (OR, 1.21; 95% CI, 1.15-1.27; *P* < .001), eclampsia (OR, 8.38; 95% CI, 7.29-9.64; *P* < .001), gestational hypertension (OR, 1.16; 95% CI, 1.09-1.23; *P* < .001), hypertension (OR, 1.67; 95% CI, 1.59-1.75; *P* < .01), asthma (OR, 1.10; 95% CI, 1.07-1.14; *P* <.001), obesity (OR, 1.16; 95% CI, 1.12-1.20; *P* < .001), premature birth (OR, 1.05; 95% CI, 1.00-1.10; *P* < .05), severe maternal morbidity (OR, 2.57; 95% CI, 2.44-2.70; *P* < .001), and blood products transfusion (OR, 1.43; 95% CI, 2.44-2.70; *P* < .001). Complications associated with lower odds of postpartum ED visits were multiple gestations (OR, 0.93; 95% CI, 0.87-1.00; *P* = .05), prior cesarean birth (OR, 0.86; 95% CI, 0.83-0.88; *P* < .001), and gestational diabetes (OR, 0.96; 95% CI, 0.92-1.00; *P* > .05).

Birth at safety net hospitals (OR, 1.43; 95% CI, 1.37-1.51; *P* < .001), minority-serving hospitals (OR, 1.14; 95% CI, 1.08-1.20; *P* < .001), and at hospitals with fewer than 500 births per year (OR, 1.25; 95% CI, 1.14-1.39; *P* < .001) relative to those with 2001 or more annual births were associated with higher adjusted odds of postpartum ED visits. Birth at a teaching hospital (OR, 0.82; 95% CI, 0.74-0.91; *P* < .001) or in a metropolitan area (OR, 0.74; 95% CI, 0.65-0.85; *P* < .001) was associated with lower adjusted odds of postpartum ED visits. The adjusted odds of postpartum ED visits decreased as the ratio of obstetric to total births increased (OR, 0.93; 95% CI, 0.91-0.94; *P* = .01).

The 3 most common primary *ICD-9* and *ICD-10* diagnosis codes associated with ED visits after obstetric discharge were nonspecific descriptors for postpartum complications (eAppendix 1 in Supplement 1). This was followed by *ICD* codes for postpartum hemorrhage, infections, headaches, cesarean birth wound complications, hypertensive disorders, and abnormal uterine bleeding.

## Discussion

From 2014 to 2016, nearly 1 in 15 of patients who gave birth in New York State visited an ED within 42 days. Our findings contribute to the literature by characterizing hospital-level factors associated with postpartum ED visit rates: patients who gave birth at minority-serving, safety net, and low obstetric volume facilities were disproportionately more likely to experience postpartum ED visits, while Black and Hispanic patients were more likely to have given birth at these types of hospitals. Across nearly all hospital types, Black patients experienced the highest rate of postpartum ED visits. Patients of younger age, who were Black or Hispanic, had public insurance, comorbidities, obstetric complications, and those who gave birth at safety net and minority-serving hospitals were more likely to experience postpartum ED visits.

Our findings suggest an opportunity to improve care coordination at hospitals with the highest postpartum ED visit rates. Specifically, we found that postpartum ED visits are not equally distributed across hospitals or hospital types and that patients who give birth at minority-serving, safety net, nonmetropolitan, and low obstetric volume facilities were more likely to experience ED visits within 42 days of obstetric discharge. White patients were underrepresented at safety net and minority-serving hospitals (accounting for only 5.5% and 10.3% of obstetric discharges, respectively). While the latter is less surprising given the definition of minority-serving hospitals, White patients were also overrepresented relative to other racial and ethnic groups at nonmetropolitan hospitals and hospitals with the lowest obstetric volumes. These findings suggest differing reasons for postpartum ED visits may be at play. For example, patients giving birth at lower volume and nonmetropolitan hospitals may experience geographic barriers to accessing postpartum care regardless of race. On the other hand, those giving birth at metropolitan, safety net, and minority-serving hospitals—who are disproportionately from racial or ethnic minority groups—may experience adverse effects related to structural racism. The racial differences at safety net and minority-serving hospitals suggest future work is needed to evaluate the role of hospital-level racial segregation in maternal morbidity.

Across hospital types, we observed racial disparities in postpartum ED visit rates. Black patients had more than double the overall postpartum ED visit rate of White patients in the overall sample (9.2 vs 4.5%). They experienced the highest rates of postpartum ED visit rates across all hospital types, with the exception of nonmetropolitan hospitals, where Hispanic patients experienced the highest postpartum ED visit rates (while Black and White patients have comparable postpartum ED visit rates). In addition, we observe in Figure 2 that White and Asian patients experienced the lowest postpartum ED visit rates among racial groups at minority-serving and safety net hospitals. Furthermore, racial and ethnic differences in postpartum ED visit rates were of a lower magnitude at nonminority serving and non-safety hospitals. This suggests that within-hospital racial disparities may be particularly problematic at facilities that disproportionately serve racial and ethnic minority groups. These wider within-hospital racial disparities at safety net facilities likely represent the multifactorial effects of structural racism—Black and Hispanic pregnant people are at higher risk for obstetric complications, experience barriers to accessing care,^20^ and are more likely to give birth at safety net hospitals that have been subjected to decades of disinvestment^21^ and segregation.^22^ Further research is needed to understand how to close these racial and ethnic disparities at both structural policy and organizational levels. Previously proposed organization-level interventions include strong clinician team communication and teamwork, bias training for leadership and staff, and targeted education around bias resulting in differential treatment in hospital settings.^23^

Our findings are consistent with and extend upon prior work highlighting the role of hospital factors in obstetric outcomes, including severe maternal morbidity. Black and Hispanic patients are more likely to give birth at these hospitals, and more likely to experience higher rates of maternal morbidity compared with other racial groups giving birth at the same hospital.^24,25^ Our findings are also consistent with prior work demonstrating that patients who are younger, from racial and ethnic minority groups, publicly insured, and have more comorbid medical conditions are more likely to experience postpartum ED visits and readmissions.^4,11,26,27^ Of note, while patients from the lowest income quartiles zip codes had higher rates of postpartum ED visits, they had lower adjusted odds of postpartum ED visits after accounting for patient and hospital characteristics. These seemingly paradoxical findings may be explained by recent evidence that preterm birth and low birth weight are more frequent among high-income patients,^28^ likely due to the use of assisted reproductive technology. Our findings shed light on the hospital-level factors driving racial and ethnic disparities in postpartum acute care use and highlight the need for systemic and institution-level interventions to close between and within-hospital gaps.

### Limitations

This study had limitations. The administrative nature of the State Inpatient Database and the State Emergency Department Database lacked clinical data, such as vital signs or prenatal care, which prohibited us from controlling for various patient clinical characteristics. For example, we could not assess the regional availability of obstetric clinicians, which may play a large role in postpartum access to care, particularly in rural settings. However, we adjusted for comorbidities that are known to affect maternal outcomes. In addition, this study only examined 42 days postpartum despite the WHO^29^ and CDC recognizing postpartum morbidity and mortality through 1 year after birth. While later events could have been excluded, the time corresponds to the period when patients are most likely to experience an ED visit and to guidelines in effect at the time of data collection. Overall, this study is strengthened by its generalizability because it examined birth records across an entire state. However, it may not be generalizable to the United States as a whole, which is vastly diverse in terms of health care needs and access to hospitals.

## Conclusions

The findings of this cross-sectional study suggest that postpartum ED visits are more frequent among groups that have been economically and socially marginalized. These patients are also at the highest risk for maternal morbidity and mortality. Within-hospital racial and ethnic disparities and between-hospital differences in the racial and ethnic patient population at various hospital types appear to account for substantial differences in postpartum ED visits. Improved care coordination for high-risk patients and system-level interventions to mitigate structural racism is needed to prevent avoidable ED visits and improve outcomes during this critical period. Hospital- and system-level efforts to improve care coordination and reduce postpartum ED visits have the potential to improve maternal morbidity and mitigate racial and ethnic differences in obstetric outcomes.

## Notes

Supplement 1.
eAppendix.

Supplement 2.
Data Sharing Statement

## References

[1] Centers for Disease Control and Prevention. Maternal mortality rates in the United States, 2020. Accessed February 14, 2023. https://www.cdc.gov/nchs/data/hestat/maternal-mortality/2020/maternal-mortality-rates-2020.htm

[2] Centers for Disease Control and Prevention. How does the CDC identify severe maternal morbidity? Accessed April 30, 2021. https://www.cdc.gov/reproductivehealth/maternalinfanthealth/smm/severe-morbidity-ICD.htm

[3] Harrell T, Howell EA, Balbierz A, . Improving postpartum care: identifying opportunities to reduce postpartum emergency room visits among publicly insured women of color. Matern Child Health J. 2022;26(4):913-922. doi:10.1007/s10995-021-03282-5 34982328 PMC8724640

[4] Clark SL, Belfort MA, Dildy GA, . Emergency department use during the postpartum period: implications for current management of the puerperium. Am J Obstet Gynecol. 2010;203(1):38.e1-38.e6. doi:10.1016/j.ajog.2010.02.033 20417492

[5] Brousseau EC, Danilack V, Cai F, Matteson KA. Emergency department visits for postpartum complications. J Womens Health (Larchmt). 2018;27(3):253-257. doi:10.1089/jwh.2016.6309 28937843 PMC5865248

[6] Belfort MA, Clark SL, Saade GR, . Hospital readmission after delivery: evidence for an increased incidence of nonurogenital infection in the immediate postpartum period. Am J Obstet Gynecol. 2010;202(1):35.e1-35.e7. doi:10.1016/j.ajog.2009.08.029 19889389

[7] Farr A, Lenz-Gebhart A, Einig S, . Outcomes and trends of peripartum maternal admission to the intensive care unit. Wien Klin Wochenschr. 2017;129(17-18):605-611. doi:10.1007/s00508-016-1161-z 28101669 PMC5599431

[8] Pollock W, Rose L, Dennis CL. Pregnant and postpartum admissions to the intensive care unit: a systematic review. Intensive Care Med. 2010;36(9):1465-1474. doi:10.1007/s00134-010-1951-0 20631987

[9] Selo-Ojeme DO, Omosaiye M, Battacharjee P, Kadir RA. Risk factors for obstetric admissions to the intensive care unit in a tertiary hospital: a case-control study. Arch Gynecol Obstet. 2005;272(3):207-210. doi:10.1007/s00404-004-0695-x 15690170

[10] Varner CE, Park AL, Ray JG. Prepregnancy emergency department use and risks of severe maternal and neonatal morbidity in Canada. JAMA Netw Open. 2022;5(9):e2229532. doi:10.1001/jamanetworkopen.2022.29532 36053536 PMC9440393

[11] Clapp MA, Little SE, Zheng J, Robinson JN. A multi-state analysis of postpartum readmissions in the United States. Am J Obstet Gynecol. 2016;215(1):113.e1-113.e10. doi:10.1016/j.ajog.2016.01.174 27829570

[12] Kuklina EV, Whiteman MK, Hillis SD, . An enhanced method for identifying obstetric deliveries: implications for estimating maternal morbidity. Matern Child Health J. 2008;12(4):469-477. doi:10.1007/s10995-007-0256-6 17690963

[13] Sarayani A, Wang X, Thai TN, Albogami Y, Jeon N, Winterstein AG. Impact of the transition from ICD-9-CM to ICD-10-CM on the identification of pregnancy episodes in US health insurance claims data. Clin Epidemiol. 2020;12(I2):1129-1138. doi:10.2147/CLEP.S269400 33116906 PMC7571578

[14] The American College of Obstetricians and Gynecologists. ACOG redesigns postpartum care. Accessed July 24, 2022. https://www.acog.org/news/news-releases/2018/04/acog-redesigns-postpartum-care

[15] National Heart, Lung, and Lung Institute. Systemic racism, a key risk factor for maternal death and illness. Accessed July 24, 2022. https://www.nhlbi.nih.gov/news/2021/systemic-racism-key-risk-factor-maternal-death-and-illness

[16] New York State Department of Health. Facility race/ethnicity concordance reports. Accessed February 1, 2023. https://www.health.ny.gov/statistics/sparcs/reports/race_eth/

[17] Wheeler SM, Bryant AS, Bonney EA, Howell EA; Society for Maternal-Fetal Medicine. Society for maternal-fetal medicine special statement: race in maternal-fetal medicine research: dispelling myths and taking an accurate, antiracist approach. Am J Obstet Gynecol. 2022;226(4):B13-B22. doi:10.1016/j.ajog.2021.11.023 34774520

[18] Agency for Healthcare Research and Quality. Race, ethnicity, and language data: standardization for health care quality improvement. Accessed July 24, 2022. https://www.ahrq.gov/research/findings/final-reports/iomracereport/reldata3.html

[19] Snowden JM, Lyndon A, Kan P, El Ayadi A, Main E, Carmichael SL. Severe maternal morbidity: a comparison of definitions and data sources. Am J Epidemiol. 2021;190(9):1890-1897. doi:10.1093/aje/kwab077 33755046 PMC8579027

[20] Institute of Medicine. America’s Health Care Safety Net: Intact but Endangered. The National Academies Press; 2000. doi:10.17226/9612. 25077222

[21] Smith DB. Eliminating disparities in treatment and the struggle to end segregation. Commonwealth Fund. Accessed February 14, 2023. https://www.commonwealthfund.org/publications/fund-reports/2005/aug/eliminating-disparities-treatment-and-struggle-end-segregation

[22] Lasser KE, Liu Z, Lin MY, Paasche-Orlow MK, Hanchate A. Changes in hospitalizations at US safety-net hospitals following Medicaid expansion. JAMA Netw Open. 2021;4(6):e2114343. doi:10.1001/jamanetworkopen.2021.14343 34191000 PMC8246310

[23] Howell EA, Sofaer S, Balbierz A, Kheyfets A, Glazer KB, Zeitlin J. Distinguishing high-performing from low-performing hospitals for severe maternal morbidity: a focus on quality and equity. Obstet Gynecol. 2022;139(6):1061-1069. doi:10.1097/AOG.0000000000004806 35675603 PMC9710203

[24] Howell EA, Egorova NN, Janevic T, . Race and ethnicity, medical insurance, and within-hospital severe maternal morbidity disparities. Obstet Gynecol. 2020;135(2):285-293. doi:10.1097/AOG.0000000000003667 31923076 PMC7117864

[25] Howell EA, Egorova NN, Balbierz A, Zeitlin J, Hebert PL. Site of delivery contribution to Black-White severe maternal morbidity disparity. Am J Obstet Gynecol. 2016;215(2):143-152. doi:10.1016/j.ajog.2016.05.007 27179441 PMC4967380

[26] Wang CY, Yee LM, Feinglass JM. Delivery complications and postpartum hospital use in California. Womens Health Issues. 2022;32(1):57-66. doi:10.1016/j.whi.2021.08.004 34580022 PMC8688289

[27] Allen EM, Call KT, Beebe TJ, McAlpine DD, Johnson PJ. Barriers to care and healthcare utilization among the publicly insured. Med Care. 2017;55(3):207-214. doi:10.1097/MLR.0000000000000644 27579910 PMC5309146

[28] Kennedy-Moulton K, Miller S, Persson P, Rossin-Slater M, Wherry L, Aldana G. Maternal and infant health inequality: new evidence from linked administrative data. National Bureau of Economic Research. 2022. Accessed February 14, 2023. https://www.nber.org/papers/w30693.

[29] World Health Organization. Maternal deaths. Accessed July 24, 2022. https://www.who.int/data/gho/indicator-metadata-registry/imr-details/4622

