# Supplementary material for: Patient Sociodemographics and Comorbidities and Birth Hospital Characteristics Associated With Postpartum Emergency Department Care
Source: JAMA Netw Open. 2023 Mar 21;6(3):e233927. doi: 10.1001/jamanetworkopen.2023.3927 (PMC10031389; doi:10.1001/jamanetworkopen.2023.3927)
Supplement: Supplement 1. — eAppendix. [file jamanetwopen-e233927-s001.pdf]

Supplemental Online Content

Zarrin H, Vargas-Torres C, Janevic T, Stern T, Lin MP. Patient sociodemographics and comorbidities and birth hospital characteristics associated with postpartum emergency department care. *JAMA Netw Open*. 2023;6(3):e233927. doi:10.1001/jamanetworkopen.2023.3927

eAppendix. Most Common Diagnoses Associated with ED Visits after Obstetric Discharge, New York State, 2014-2016

This supplemental material has been provided by the authors to give readers additional information about their work.

© 2023 Zarrin H et al. *JAMA Network Open.*

eAppendix. Most Common Diagnoses Associated with ED Visits after Obstetric Discharge, New York State, 2014-2016

	**Diagnosis (ICD-9 Code) from January 1, 2014 - Sept 30, 2015**	**Total ED Visits**	**Percent %**	**Diagnosis (ICD10 code) from October 1st, 2015 - Dec 31, 2016**	**Total ED Visits**	**Percent %**
**1**	Other current conditions classifiable elsewhere of mother, postpartum condition or complication (648.94)	1300	4.0	Other complications of the puerperium, not elsewhere classified (O90.89)	1466	4.5
**2**	Other complications of obstetrical surgical wounds, postpartum condition or complication (674.34)	1043	3.2	Other specified diseases and conditions complicating pregnancy, childbirth and the puerperium (O99.89)	489	1.5
**3**	Other complications of puerperium, postpartum condition or complication (674.84)	653	2.0	Infection of obstetric surgical wound (O86.0)	437	1.3
**4**	Delayed and secondary postpartum hemorrhage, postpartum condition or complication (666.24)	586	1.8	Delayed and secondary postpartum hemorrhage (O72.2)	313	1.0
**5**	Infections of genitourinary tract in pregnancy, postpartum condition or complication (646.64)	544	1.7	Urinary tract infection, site not specified (N39.0)	307	0.9
**6**	Infection of kidney (599.0)	529	1.6	Endometritis following delivery (O86.12)	287	0.9
**7**	Headache (784.0)	444	1.4	Disruption of cesarean delivery wound (O90.0)	271	0.8
**8**	Other specified complications of pregnancy, postpartum condition or complication (646.84)	428	1.3	Headache (R51)	252	0.8
**9**	Mild or unspecified pre-eclampsia, postpartum condition or complication (642.44)	375	1.1	Eclampsia complicating the puerperium (O15.2)	233	0.7
**10**	Disruption of cesarean wound, postpartum condition or complication (674.414)	373	1.1	Abnormal uterine and vaginal bleeding, unspecified (N93.9)	210	0.6

© 2023 Zarrin H et al. *JAMA Network Open.*
