# Supplementary material for: Patient Sociodemographics and Comorbidities and Birth Hospital Characteristics Associated With Postpartum Emergency Department Care
Source: JAMA Netw Open. 2023 Mar 21;6(3):e233927. doi: 10.1001/jamanetworkopen.2023.3927 (PMC10031389; doi:10.1001/jamanetworkopen.2023.3927)
Supplement: Supplement 2. — Data Sharing Statement [file jamanetwopen-e233927-s002.pdf]

Data Sharing Statement

Zarrin. Patient Sociodemographics and Comorbidities and Birth Hospital Characteristics Associated With Postpartum Emergency Department Care. *JAMA Netw Open*. Published March 21, 2023. doi:10.1001/jamanetworkopen.2023.3927

Data
Data available: No

Additional Information
Explanation for why data not available: The data is already publicly available.
